# Supplementary material for: High-Throughput Screening for Inhibitors of the SARS-CoV-2 Protease Using a FRET-Biosensor
Source: Molecules. 2020 Oct 13;25(20):4666. doi: 10.3390/molecules25204666 (PMC7587356; doi:10.3390/molecules25204666)
Supplement: Supplementary file 1 [file molecules-25-04666-s001.zip › Supplementary_proofed_plainText.docx]

**Supplementary Figure S1:** Codon-optimized eCFP-Venus biosensor expressed from pET28a. The sequence from the start codon to stop codon is displayed along with the translated protein sequence. The sequences encoding eCFP (teal), the 3CL^pro^ cleavage site (grey), Venus (yellow) and His_6_-tag (orange) are highlighted.


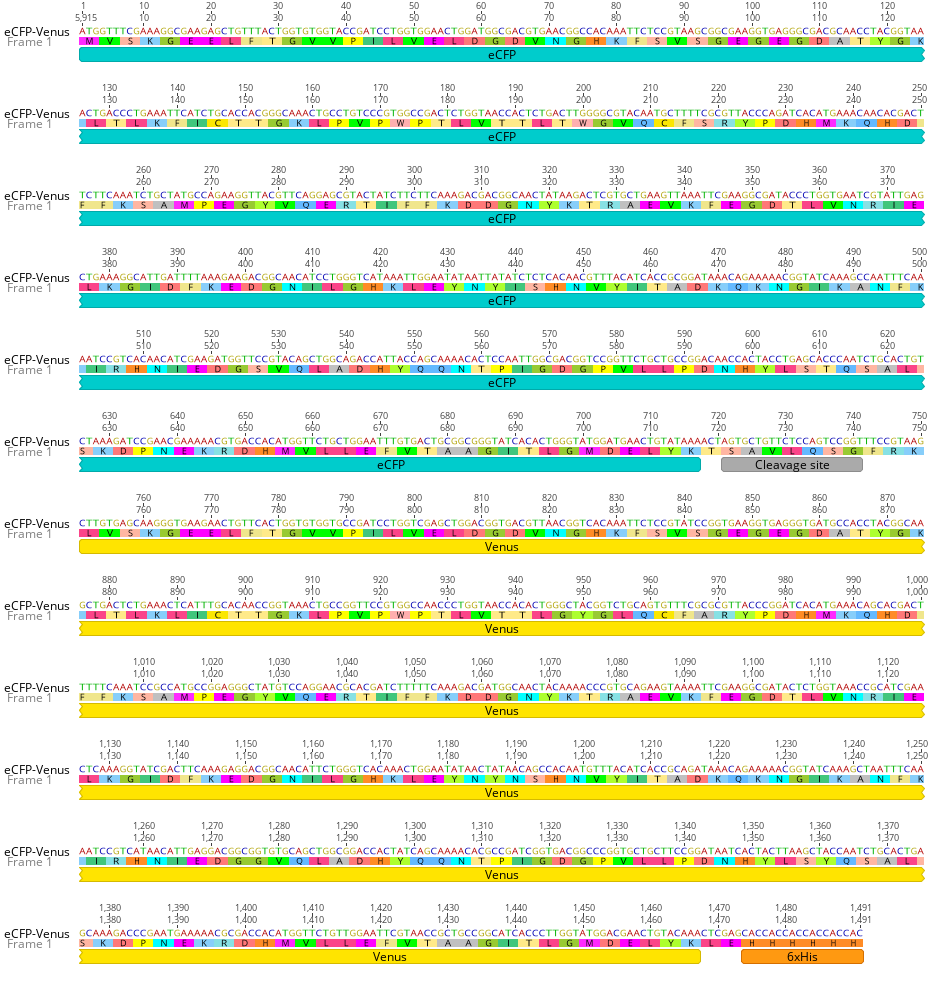


**Supplementary Figure S2:** Plate map showing controls for high-throughput screening. The LOPAC®^1280^ compounds were spread across sixteen 96-well plates leaving the left and right columns empty. The left-hand column was used for 100% inhibition controls containing no protease, i.e., none of the eCFP-Venus construct should be cut, and the right-hand column was used for 0% inhibition controls containing protease and no added compounds, i.e., no inhibitors present.


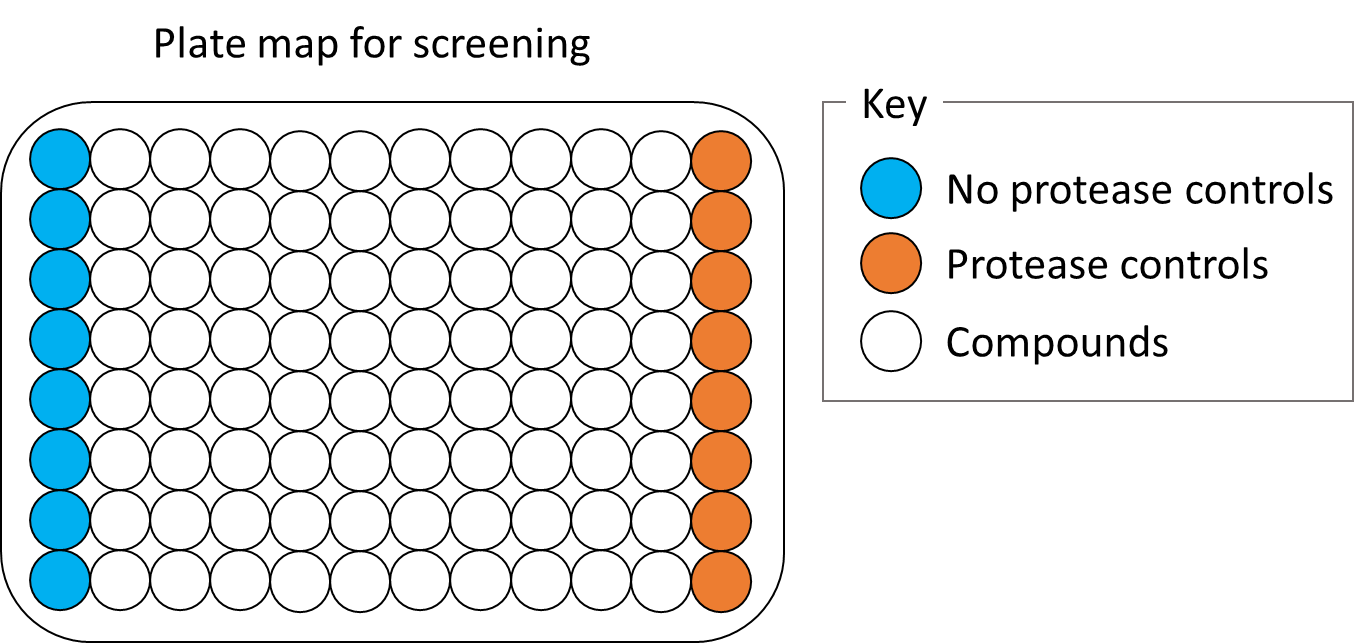


**Supplementary Figure S3:** Dose–response curves used to calculate the EC_50_ values reported in Supplementary Table S1. Assays were performed both with (blue) and without (red) 0.01% Triton X-100 as per Figure 3 of the main text. Each sample has been numbered, and the full names of the compounds tested in each cell are provided in Supplementary Table 1.


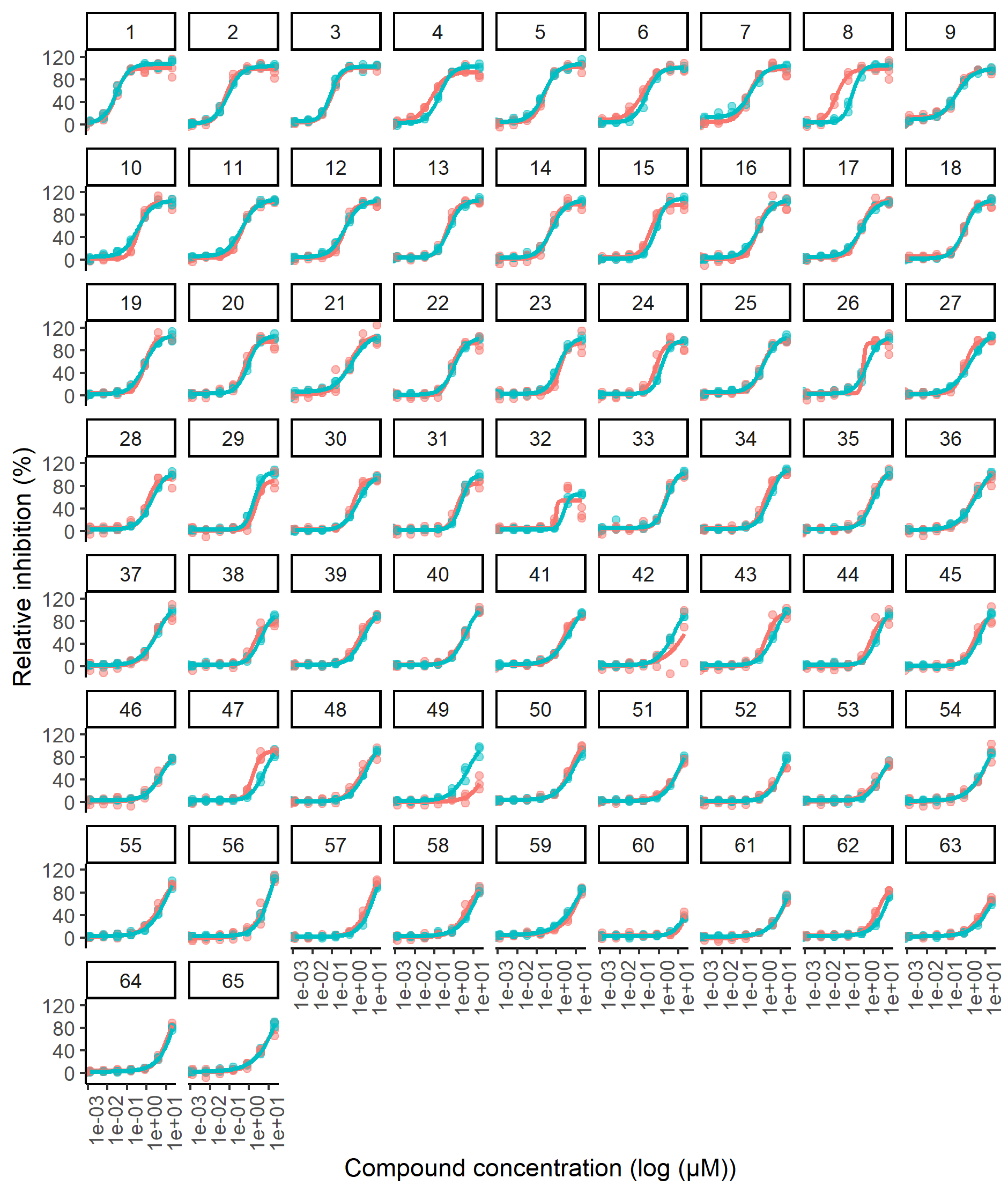


**Supplementary Table S1:** EC_50_ values of the top 65 compounds identified during screening. The column labelled “#” lists the numbers corresponding to the dose response curves shown in Supplementary Figure S3. Values were calculated for the assay repeated both with (A) and without (B) 0.01% Triton X-100. Assays were performed in triplicate and EC_50_ values were calculated separately for each replicate. The mean and standard deviation are shown. The ratio of A and B is provided to aid in identification of putative aggregation-based inhibitors, which tend to have a ratio greater than 2.

| # | Name | A) EC_50_ with Triton X-100 (µM) | B) EC_50_ without Triton X-100 (µM) | Ratio (A/B) |
| --- | --- | --- | --- | --- |
| 1 | Ebselen | 0.027 ± 0.006 | 0.026 ± 0.009 | 1.1 |
| 2 | PD 404,182 | 0.081 ± 0.009 | 0.060 ± 0.012 | 1.4 |
| 3 | 4-Chloromercuribenzoic acid | 0.085 ± 0.011 | 0.095 ± 0.007 | 0.9 |
| 4 | Disulfiram | 0.181 ± 0.021 | 0.068 ± 0.012 | 2.7 |
| 5 | alpha-NETA | 0.227 ± 0.014 | 0.243 ± 0.051 | 0.9 |
| 6 | ZPCK | 0.234 ± 0.085 | 0.136 ± 0.021 | 1.7 |
| 7 | Nordihydroguaiaretic acid | 0.270 ± 0.058 | 0.214 ± 0.065 | 1.3 |
| 8 | SCH-202676 | 0.274 ± 0.073 | 0.041 ± 0.008 | 6.7 |
| 9 | ZM 39923 | 0.302 ± 0.038 | 0.289 ± 0.055 | 1.0 |
| 10 | *R*(−)-*N*-Allylnorapomorphine | 0.346 ± 0.080 | 0.379 ± 0.083 | 0.9 |
| 11 | *R*(−)-NPA | 0.426 ± 0.017 | 0.426 ± 0.077 | 1.0 |
| 12 | *R*(−)-2-Hydroxyapomorphine | 0.450 ± 0.078 | 0.420 ± 0.101 | 1.1 |
| 13 | Apomorphine | 0.553 ± 0.076 | 0.435 ± 0.100 | 1.3 |
| 14 | Piceatannol | 0.569 ± 0.075 | 0.455 ± 0.113 | 1.3 |
| 15 | U-73122 | 0.694 ± 0.082 | 0.350 ± 0.017 | 2.0 |
| 16 | Dihydrexidine | 0.705 ± 0.153 | 0.622 ± 0.072 | 1.1 |
| 17 | *R*(−)-2-OH-NPA | 0.746 ± 0.091 | 0.715 ± 0.133 | 1.0 |
| 18 | Bromoenol lactone | 0.817 ± 0.121 | 0.757 ± 0.073 | 1.1 |
| 19 | Myricetin | 0.819 ± 0.171 | 0.750 ± 0.052 | 1.1 |
| 20 | 6-Nitroso-1,2-benzopyrone | 0.882 ± 0.110 | 0.559 ± 0.123 | 1.6 |
| 21 | Capsazepine | 0.944 ± 0.098 | 0.668 ± 0.330 | 1.4 |
| 22 | (−)-Eseroline | 0.994 ± 0.197 | 0.738 ± 0.201 | 1.3 |
| 23 | APDC | 1.234 ± 0.064 | 1.525 ± 0.697 | 0.8 |
| 24 | 6-Hydroxy-dl-DOPA | 1.317 ± 0.118 | 0.652 ± 0.259 | 2.0 |
| 25 | (±)-SKF-38393 | 1.374 ± 0.335 | 1.066 ± 0.233 | 1.3 |
| 26 | (±)-6-Chloro-PB | 1.441 ± 0.353 | 1.023 ± 0.289 | 1.4 |
| 27 | Bay 11-7085 | 1.616 ± 0.103 | 0.807 ± 0.120 | 2.0 |
| 28 | TPCK | 1.741 ± 0.034 | 0.985 ± 0.175 | 1.8 |
| 29 | Sanguinarine | 1.780 ± 0.497 | 2.241 ± 0.586 | 0.8 |
| 30 | Benserazide | 2.114 ± 0.303 | 1.189 ± 0.202 | 1.8 |
| 31 | *S*-Nitrosoglutathione | 2.294 ± 0.245 | 1.494 ± 0.627 | 1.5 |
| 32 | Aurintricarboxylic acid | 2.341 ± 0.513 | 0.901 ± 0.045 | 2.6 |
| 33 | 6,7-ADTN | 2.381 ± 0.257 | 2.123 ± 0.727 | 1.1 |
| 34 | *N*-Acetyldopamine | 2.652 ± 0.550 | 1.446 ± 0.222 | 1.8 |
| 35 | DOPAC | 3.094 ± 0.226 | 2.461 ± 0.117 | 1.3 |
| 36 | *N*-allyl-(±)-SKF 38393 | 3.342 ± 0.805 | 1.652 ± 0.462 | 2.0 |
| 37 | SKF 89626 | 3.530 ± 0.565 | 2.829 ± 1.975 | 1.2 |
| 38 | Me-3,4-dephostatin | 3.777 ± 0.867 | 1.825 ± 0.925 | 2.1 |
| 39 | A-77636 | 3.787 ± 0.630 | 2.342 ± 0.783 | 1.6 |
| 40 | Pyrocatechol | 4.045 ± 1.425 | 4.093 ± 1.222 | 1.0 |
| 41 | l-Leucinethiol | 4.377 ± 0.770 | 1.946 ± 0.110 | 2.2 |
| 42 | (±)-Pindobind | 4.693 ± 3.322 | > 20 | ND |
| 43 | Hispidin | 5.002 ± 0.554 | 1.807 ± 0.828 | 2.8 |
| 44 | Dephostatin | 5.030 ± 1.216 | 2.284 ± 0.619 | 2.2 |
| 45 | nor-Binaltorphimine | 5.205 ± 1.159 | 2.604 ± 1.493 | 2.0 |
| 46 | 3-Morpholinosydnonimine | 5.656 ± 0.445 | 4.966 ± 2.380 | 1.1 |
| 47 | Lansoprazole | 5.715 ± 0.865 | 1.462 ± 0.398 | 3.9 |
| 48 | GED | 5.830 ± 0.664 | 3.640 ± 2.427 | 1.6 |
| 49 | 1-Hbit | 7.077 ± 5.153 | > 20 | ND |
| 50 | 3,4-Dichloroisocoumarin | 8.698 ± 2.720 | 5.379 ± 0.612 | 1.6 |
| 51 | TLCK | 10.633 ± 0.881 | > 20 | ND |
| 52 | Cisplatin | 14.476 ± 4.689 | > 20 | ND |
| 53 | Diamide | 17.063 ± 20.824 | > 20 | ND |
| 54 | SKF 75670 | 17.776 ± 8.363 | > 20 | ND |
| 55 | Tyrphostin 51 | 19.003 ± 14.172 | 9.122 ± 5.296 | 2.1 |
| 56 | Idarubicin | > 20 | > 20 | ND |
| 57 | Tyrphostin 47 | > 20 | 15.773 ± 10.143 | ND |
| 58 | Fenoldopam | > 20 | 7.187 ± 6.083 | ND |
| 59 | Caffeic acid phenethyl ester | > 20 | > 20 | ND |
| 60 | CPCCOEt | > 20 | > 20 | ND |
| 61 | NSC 95397 | > 20 | > 20 | ND |
| 62 | Caffeic Acid | > 20 | 5.388 ± 1.226 | ND |
| 63 | (+)-Catechin | > 20 | 11.308 ± 6.553 | ND |
| 64 | *R*(+)-6-Bromo-APB | > 20 | > 20 | ND |
| 65 | SKF 83565 | > 20 | 6.031 ± 3.63 | ND |

**Supplementary Sequence S1:** Codon-optimized eCFP-Venus with His_6_-tag. The underlined region is part of the *Nco*I restriction site of the vector.

ATGGTTTCGAAAGGCGAAGAGCTGTTTACTGGTGTGGTACCGATCCTGGTGGAACTGGATGGCGACGTGAACGGCCACAAATTCTCCGTAAGCGGCGAAGGTGAGGGCGACGCAACCTACGGTAAACTGACCCTGAAATTCATCTGCACCACGGGCAAACTGCCTGTCCCGTGGCCGACTCTGGTAACCACTCTGACTTGGGGCGTACAATGCTTTTCGCGTTACCCAGATCACATGAAACAACACGACTTCTTCAAATCTGCTATGCCAGAAGGTTACGTTCAGGAGCGTACTATCTTCTTCAAAGACGACGGCAACTATAAGACTCGTGCTGAAGTTAAATTCGAAGGCGATACCCTGGTGAATCGTATTGAGCTGAAAGGCATTGATTTTAAAGAAGACGGCAACATCCTGGGTCATAAATTGGAATATAATTATATCTCTCACAACGTTTACATCACCGCGGATAAACAGAAAAACGGTATCAAAGCCAATTTCAAAATCCGTCACAACATCGAAGATGGTTCCGTACAGCTGGCAGACCATTACCAGCAAAACACTCCAATTGGCGACGGTCCGGTTCTGCTGCCGGACAACCACTACCTGAGCACCCAATCTGCACTGTCTAAAGATCCGAACGAAAAACGTGACCACATGGTTCTGCTGGAATTTGTGACTGCGGCGGGTATCACACTGGGTATGGATGAACTGTATAAAACTAGTGCTGTTCTCCAGTCCGGTTTCCGTAAGCTTGTGAGCAAGGGTGAAGAACTGTTCACTGGTGTGGTGCCGATCCTGGTCGAGCTGGACGGTGACGTTAACGGTCACAAATTCTCCGTATCCGGTGAAGGTGAGGGTGATGCCACCTACGGCAAGCTGACTCTGAAACTCATTTGCACAACCGGTAAACTGCCGGTTCCGTGGCCAACCCTGGTAACCACACTGGGCTACGGTCTGCAGTGTTTCGCGCGTTACCCGGATCACATGAAACAGCACGACTTTTTCAAATCCGCCATGCCGGAGGGCTATGTCCAGGAACGCACGATCTTTTTCAAAGACGATGGCAACTACAAAACCCGTGCAGAAGTAAAATTCGAAGGCGATACTCTGGTAAACCGCATCGAACTCAAAGGTATCGACTTCAAAGAGGACGGCAACATTCTGGGTCACAAACTGGAATATAACTATAACAGCCACAATGTTTACATCACCGCAGATAAACAGAAAAACGGTATCAAAGCTAATTTCAAAATCCGTCATAACATTGAGGACGGCGGTGTGCAGCTGGCGGACCACTATCAGCAAAACACGCCGATCGGTGACGGCCCGGTGCTGCTTCCGGATAATCACTACTTAAGCTACCAATCTGCACTGAGCAAAGACCCGAATGAAAAACGCGACCACATGGTTCTGTTGGAATTCGTAACCGCTGCCGGCATCACCCTTGGTATGGACGAACTGTACAAA

**Supplementary Sequence S2:** Codon-optimized SARS-CoV-2 3CL^pro^. The underlined region is part of the *Nco*I restriction site of the vector.

ATGGGCACGAGCGCGGTTCTGCAATCAGGTTTCCGTAAAATGGCTTTCCCGAGCGGCAAAGTGGAAGGCTGCATGGTTCAGGTGACCTGCGGTACGACTACTCTGAATGGCCTGTGGCTCGACGACGTTGTCTACTGCCCGCGTCACGTTATTTGTACCAGCGAAGATATGCTGAACCCGAATTACGAAGACCTGCTGATTCGCAAATCCAACCACAACTTCCTGGTTCAGGCCGGTAACGTTCAACTGCGCGTAATCGGCCACTCTATGCAGAACTGCGTACTGAAGCTGAAAGTTGATACGGCGAACCCGAAAACTCCGAAATACAAGTTCGTGCGTATCCAGCCTGGCCAAACTTTCTCTGTTCTCGCTTGTTATAACGGTAGCCCGTCTGGCGTTTACCAGTGTGCTATGCGTCCAAACTTCACAATCAAAGGCTCCTTCCTGAACGGCTCGTGCGGCTCTGTCGGTTTCAACATCGACTATGATTGCGTTTCTTTTTGCTATATGCATCACATGGAGCTGCCGACTGGTGTACACGCGGGTACGGACCTGGAAGGTAACTTCTACGGTCCGTTCGTTGATCGCCAGACCGCCCAGGCAGCTGGTACTGATACCACTATCACCGTCAATGTCCTGGCTTGGCTGTACGCTGCGGTGATCAACGGTGATCGCTGGTTCCTGAACCGTTTCACTACGACCCTGAACGACTTCAACCTGGTGGCTATGAAGTATAACTATGAACCGCTGACTCAGGATCATGTAGATATTCTGGGCCCGCTGTCGGCACAAACTGGCATTGCGGTGCTGGATATGTGTGCGAGCCTGAAGGAACTGCTGCAGAACGGTATGAATGGCCGCACTATCCTGGGTAGCGCGCTGCTGGAGGATGAGTTCACCCCGTTTGACGTTGTTCGTCAGTGTTCCGGTGTTACCTTCCAGGGTCCGCACCATCACCATCACCATTAA
